# Options for the generation of seedless cherry, the ultimate snacking product

**DOI:** 10.1007/s00425-022-04005-y

**Published:** 2022-09-28

**Authors:** Edoardo Vignati, Marzena Lipska, Jim M. Dunwell, Mario Caccamo, Andrew J. Simkin

**Affiliations:** 1 NIAB East Malling, Department of Genetics, Genomics and Breeding, New Road, West Malling, Kent ME19 6BJ UK; 2 School of Agriculture, Policy and Development, University of Reading, Whiteknights, Reading, Berkshire RG6 6EU UK; 3 NIAB, Cambridge Crop Research, Lawrence Weaver Road, Cambridge, CB3 0LE UK; 4 School of Biosciences, University of Kent, Canterbury, CT2 7NJ UK

**Keywords:** Parthenocarpy, Fruit, Cherry, Seed, June drop

## Abstract

**Main conclusion:**

This manuscript identifies cherry orthologues of genes implicated in the development of pericarpic fruit and pinpoints potential options and restrictions in the use of these targets for commercial exploitation of parthenocarpic cherry fruit.

**Abstract:**

Cherry fruit contain a large stone and seed, making processing of the fruit laborious and consumption by the consumer challenging, inconvenient to eat ‘on the move’ and potentially dangerous for children. Availability of fruit lacking the stone and seed would be potentially transformative for the cherry industry, since such fruit would be easier to process and would increase consumer demand because of the potential reduction in costs. This review will explore the background of seedless fruit, in the context of the ambition to produce the first seedless cherry, carry out an in-depth analysis of the current literature around parthenocarpy in fruit, and discuss the available technology and potential for producing seedless cherry fruit as an ‘ultimate snacking product’ for the twenty-first century.

## Introduction

Cherry (*Prunus avium*) trees are believed to be native to Europe, south Asia and to an isolated region of the western Himalaya (Faust and Surányi 1997). Commonly known as sweet cherry, it is a deciduous tree belonging to the Rosaceae family (https://sweetbiomics.com/) (Ganopoulou et al. 2022), which also includes apple (*Malus x domestica*), peach (*Prunus persica*), and strawberry (*Fragaria vesca* and *F. ananassa*). Young cherry trees show strong apical dominance with a straight trunk which grows to between 15 and 32 m in height. The species has become naturalized in North America and Australia, since it is largely cultivated in these regions due to its commercial importance.

The sweet cherry fruit is a drupe, 1–2 cm in diameter with an attractive appearance, due to its colour (bright red to dark purple) and pleasant intense flavour. The fruit are also nutrient dense, high in fibre and contain a large range of bioactive compounds including polyphenols, anthocyanins, carotenoids, and vitamins (Kelley et al. 2018; McCune et al. 2011; Vignati et al. 2022). Cherries also contain high levels of minerals including magnesium (10 mg/100 g), potassium (200 mg/100 g), phosphorous (20 mg/100 g), and calcium (14 mg/100 g) (Ferretti et al. 2010). Cherries are also a good source of tryptophan and serotonin (Cubero et al. 2010; Garrido et al. 2012). These data suggest that increasing cherry consumption could be beneficial to human health and quality of life of consumers (Alba et al. 2019; Bell et al. 2014; Coelho Rabello Lima et al. 2015; Ferretti et al. 2010; Kelley et al. 2018; McCune et al. 2011;). Some of the most critical attributes desired by consumers include colour, firmness, sweetness, and flavour intensity (Cliff et al. 1996; Guyer et al. 1993; Kappel et al. 1996; Zheng et al. 2016); however, consumers from different regions set different requirements for what is considered a good cherry. For example, in Norwegian, American, and UK markets, consumers prefer larger dark cherries (Crisosto et al. 2003; Lyngstad and Sekse 1995; Sekse and Lyngstad 1996; Wermund and Fearne 2000). Growers and supermarkets often have a different set of criteria for evaluating cherry quality. Specifically, growers want cherries that are firm enough to resist damage during picking, processing, and transporting and easy to harvest (long peduncles), while supermarkets insist on a long shelf life.

**Fig. 1:**
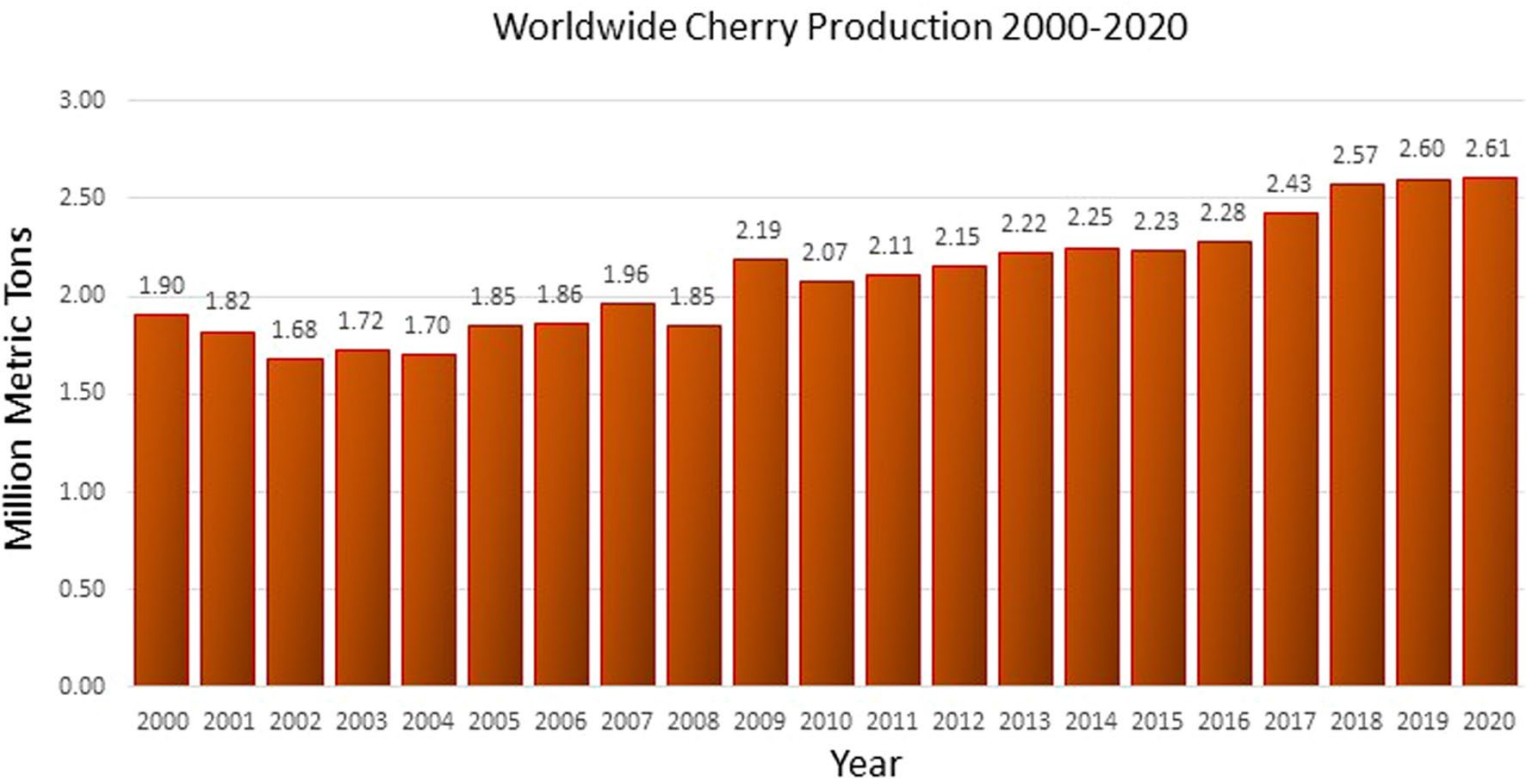
Graphical representation of worldwide cherry production between 2000 and 2020 (Statista.org). Cherry production has increased by 37% between 2000 and 2020 with the lowest production in 2002

Worldwide, cherry production has increased by almost 40% over the last 20 years as the fruit becomes more popular with consumers (Fig. 1). However, cherry fruit contains a large stone and seed, the presence of which makes it a potential choking hazard for small children and inconvenient to eat ‘on the move’. Identifying a means of generating a seedless, stoneless cherry has the potential of making the fruit even more attractive to the consumer. Previous data have shown that seedless cultivars of fruit such as sweet orange (*Citrus sinensis*), grape (*Vitis vinifera*), and watermelon (*Citrullus lanatus*) have significantly increased consumer consumption (Pollack 2001). In the case of cherry, the absence of the seeds/stone would likewise increase its potential as a snacking product and would also reduce processing costs, thus improving profit margins. An interesting model for stoneless fruit has been identified in *P. domestica* (Plum).

### Stoneless plum, a retrospective

The role of fruit is to protect the embryo and seed during development and facilitate their dispersal within the environment. Normally, after fertilization of the ovules, fruits develop from the ovary, a process that is coordinated by signals from the developing embryo (Gillaspy et al. 1993). This process of seed, stone, and fruit development is controlled and synchronized by phytohormones (Gillaspy et al. 1993). Previous work, however, has demonstrated that fruit set can be uncoupled from fertilization, resulting in the formation of seedless crops (Gorguet et al. 2005; Hu et al. 2018; Varoquaux et al. 2000). A fruit is considered seedless if it is devoid of seeds, has a significant reduction in seed number, or contains a small number of aborted seeds (Varoquaux et al. 2000). Seedless fruits can be obtained by two different routes, first, parthenocarpy, where the fruit develops without fertilization (Joldersma and Liu 2018; Picarella and Mazzucato 2019) and second, stenospermocarpy, where the seeds abort following fertilization, resulting in traces of aborted seed within the fruit (Varoquaux et al. 2000). Seedlessness has been reported in many species, including grape, citrus, tomato, pear, and banana (Ding et al. 2013; Kim et al. 1992; Klap et al. 2017; Royo et al. 2018; Simmonds 1953; Talon et al. 1990; Wang et al. 2021).

An interesting model for stoneless fruit is seen in a naturally occurring plum (*P. domestica*) cultivar called “Stoneless” (or “Pitless”), a name given on the basis that it has an underdeveloped stone that makes the fruit partially stoneless. The “Stoneless” ancestor is the variety “Sans-Noyau”, which is also stoneless (its name in French means “without pit”). “Sans-Noyau” has been known in France for several hundred years and in the 1890s was transported to the U.S., where it was crossed with varieties of commercial interest to transmit the stoneless trait. However, the results were not commercially successful (Callahan et al. 2015). Compared to a stoned plum, the expression pattern of lignin biosynthetic genes is similar (Galimba et al. 2020). However, the number of cells in the endocarp has been shown to be reduced compared to the stoned control used in the study (Callahan et al. 2009, 2015). An RNA expression comparison between “Stoneless” and normal stoned cultivars showed that more than 2000 genes are differentially expressed during fruit development, although the authors did not report the identity of these genes and confirmed the necessity of further analysis to find the gene mutation responsible of the stoneless phenotype (Galimba et al. 2020). Segregation analysis suggested that the “Stoneless” trait is dominant and linked to a single gene (Callahan et al. 2015).

### Physiological changes during cherry seed development

In the cherry ovary, there are two ovules that arise from the opposite placentae of the single carpel, while in some cultivars, one of the two fertilized arrests its growth before full bloom and collapses; in other cultivars, both ovules develop, but one of the two embryos aborts.

During fertilization, one of the two sperm cells fuses with the egg cell to form the diploid zygote, 2n, and the other one fuses with the central cell, which is 2n, to form the triploid tissue called the endosperm, the function of which is to accumulate nutrients that will be used by the embryo during the seed development. At the time of fertilization, there is a rapid elongation of the megasporocyte, which is enclosed by the megaspore membrane, while after fertilization occurs, both the embryo and the endosperm develop very slowly for a considerable time. In the first stages of the endosperm development after mitosis, there is no cell wall formation, leading to the formation of a non-cellularized syncytium. Cellularization starts at the micropylar pole of the megagametophyte and proceeds progressively to the chalazal end and from the periphery inwards to the centre. The delayed development period of the endosperm is followed by a period of very rapid enlargement (Tukey 1933).

The first zygotic division is transverse and asymmetric, leading to the formation of two cells, a smaller one above a larger cell, the suspensor cell, which forms the suspensor, the function of which is to connect the embryo to the mother plant, allowing the exchanges of nutrients and signal molecules. The suspensor is small, and it persists through to maturity of the embryo. The development of the embryo is even slower than that of the endosperm at the beginning, and then, this phase is followed by a sudden rapid development. At maturity, the embryo is made by two well-formed developed cotyledons, surrounded by a thin layer of endosperm and a fine line of nucellar tissue, and everything is enclosed by the seed coat, a completely maternal tissue, since it is derived from the ovule integuments. As noted above, two embryos can be formed in some cultivars, but one of the two suddenly arrests in development with the nucellar tissue that collapses and the integuments that shrivel. The aborted embryo can either disintegrate 5 days after arrest or can remain intact for up to 42 days.

**Fig. 2:**
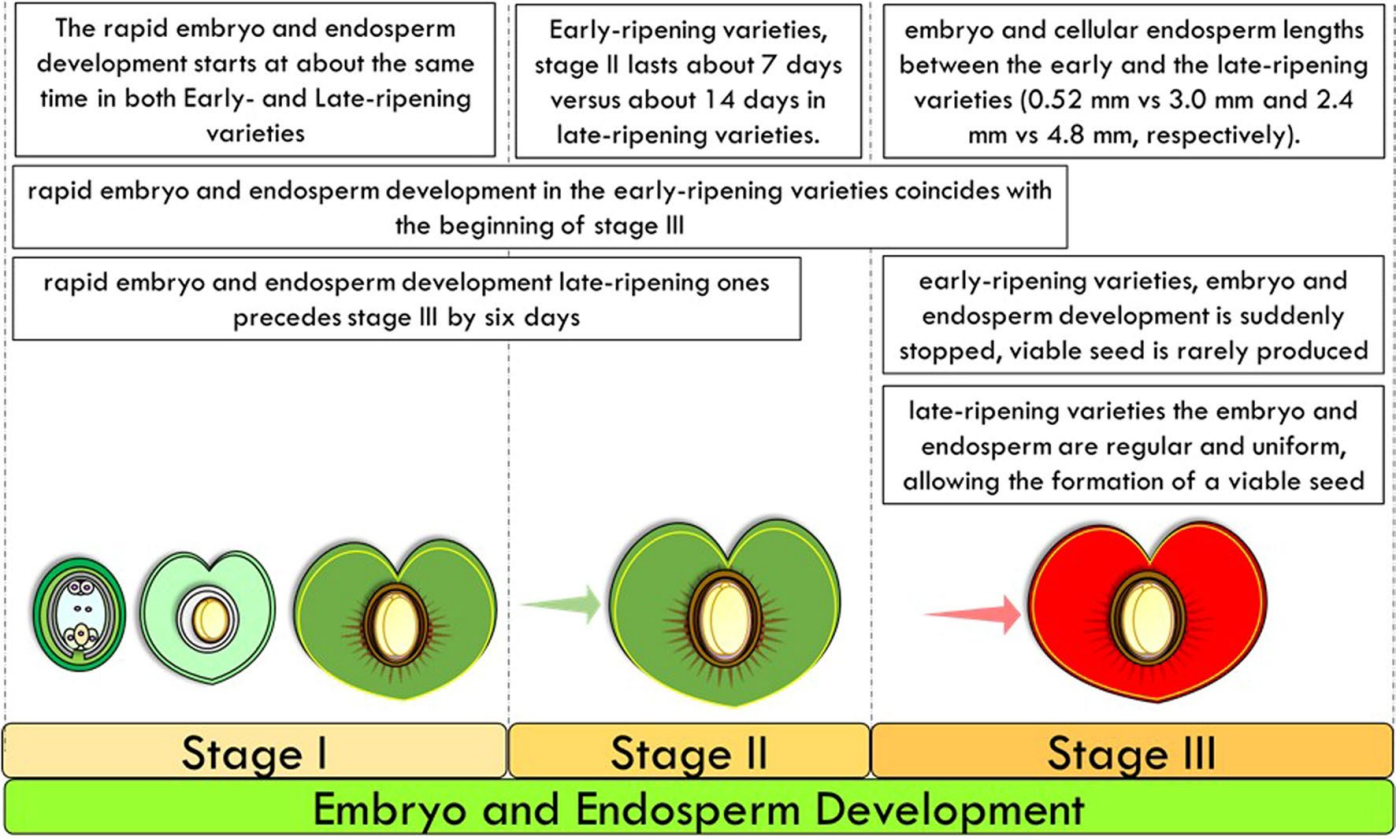
Graphical representation of embryo and endosperm development in the cultivated cherry during the three stages of fruit development

There is a wide variation in the stage of the fruit and embryo development at which abortion can occur. Seed abortion is usually associated with fruit drop (see section June drop), but it could also be a feature of mature fruits in certain varieties. For example, early ripening varieties produce only about 15% of viable seeds, while the seeds of the late-ripening varieties are nearly 100% viable (Fig. 2).

During the pre-bloom phase, both the early ripening and the late-ripening varieties develop similarly in all parts, with the only difference being that the early ripening varieties develop slightly in advance and at a slightly greater rate than the late-ripening cherries (Stage I). In early ripening varieties, stage II lasts about half the time compared to late-ripening varieties (about 7 days versus about 14 days when comparing the Early Purple Guigne and the Downer cultivars). The rapid embryo and endosperm development starts at about the same time in both types, and while in the early ripening varieties, this also coincides with the beginning of stage III; in the late-ripening ones, this precedes stage III by 6 days (Tukey 1933).

At the beginning of stage III, there is a big difference in embryo and cellular endosperm lengths between the early and the late-ripening varieties (0.52 mm vs 3.0 mm and 2.4 mm vs 4.8 mm, respectively). In early-ripening varieties, embryo and endosperm development is halted at varying stages, so a viable seed is produced only rarely, while in the late-ripening varieties, they are regular and uniform, allowing the formation of a viable seed. Indeed, the seeds formed by early-ripening varieties are shrivelled, because both the nucellus and the integuments collapse, while the seed of the late-ripening varieties are plump and well filled. Seed abortion occurs at an earlier date in the early ripening varieties, producing smaller aborted embryos, compared with those ripening a few days later. Moreover, seed abortion seems to be not necessarily associated with poor pollination, sterility, incompatibilities, or even nutrition (Tukey 1933, 1934).

### The role of hormones in seed development

#### Gibberellin is sufficient to initiate fruit set

Hormones play an essential role during all the developmental processes and it has long been known that the application of phytohormones to emasculated flowers induces the formation of parthenocarpic fruit (Sjut and Bangerth 1982). Crane et al. (1960) showed that gibberellin (GA) treatment of different plants belonging to the *Prunus* genus is sufficient to start fruit set in some of them. Indeed, while *P. domestica* (plum) and *P. avium* (sweet cherry) did not produce any parthenocarpic fruit, *P. dulcis* (almond), *P. armeniaca* (apricot), and *P. persica* (peach) developed parthenocarpic fruits with a success rate of 11.8%, 15.4% (500 ppm GA treatment), and 73.4%, respectively (50 ppm GA treatment applied twice). Although the highest fruit set was recorded in peach with the lowest concentration of GA, the highest concentration (500 ppm) led to a higher level of stone development and degree of sclerification, which is absent in the fruits treated with 50 ppm solution. The parthenocarpic peaches reached maturation about 10 days before the seeded fruit (Crane et al. 1960). It is likely that the failure to induce parthenocarpy in cherry was due to an incorrect concentration or type of GA, because Wen et al. (2019) showed that treating emasculated *P. pseudocerasus* (Chinese cherry) pistils with 300 mg/L GA_3_ solution was enough to initiate the development of parthenocarpic fruit. At 7 days after treatment (DAT), there is an upregulation of *the Gibberellin 2-beta-dioxygenase gene (PaGA2ox),* which encodes a GA biosynthetic enzyme. Moreover, a list of differentially expressed genes has been reported, and this could be used to identify those involved in the fruit set process in cherry (Wen et al. 2019). Furthermore, Galimba et al. showed that the application of GA_3_ to Honeycrisp apple flowers triggers the development of parthenocarpic apple fruit (Galimba et al. 2019).

As regards mode of action, gibberellins seem to be involved in the activation of the cell cycle in the ovary-wall cells. In fact, Mesejo et al. (2016) compared the GA levels and the gene expression in two different species of Citrus, Satsuma (*Citrus unshiu)* and the hybid clementine (*Citrus x clementina)*, which are obligate and facultative parthenocarpic, respectively. In Satsuma, the expression levels of *CYCA1.1*, which encodes for a cyclin expressed during the G2 phase and controls the G2/M phases of the cell cycle (Fabian et al. 2000), are higher than in clementine. Interestingly, in Satsuma, there is a peak of *GA20 oxidase 2 (GA20ox2)* expression just before the peak of *CYCA1.1* expression, while in clementine, this is delayed. Another GA biosynthesis-related gene, *GA3 oxidase 2*, has a higher expression level in Satsuma than clementine, during fruit development. Treatments with GA_3_ caused an increase of GA_20_ and GA_1_, which is the most important GA precursor in Satsuma ovaries. More importantly, a 132% increase of *CYCA1.1* expression has been observed 6 h after treatment, and this effect was lost after 2 days. The treatment with paclobutrazol (PBZ), an inhibitor of GA biosynthesis, caused a decrease of *CYCA1.1* expression after 6 h and this effect was maintained up to 2 days after, proving once again that GAs influence the transcription rate of *CYCA1.1*. GA_3_ enhances cell division, increasing the number of cell layers in the pericarp (effect lost at 15 DAT), while the opposite and expected effect has been observed after PBZ treatment. These effects on cell division and parthenocarpic fruit set upon GA_3_ application have also been observed in clementine (Mesejo et al. 2016).

**Fig. 3:**
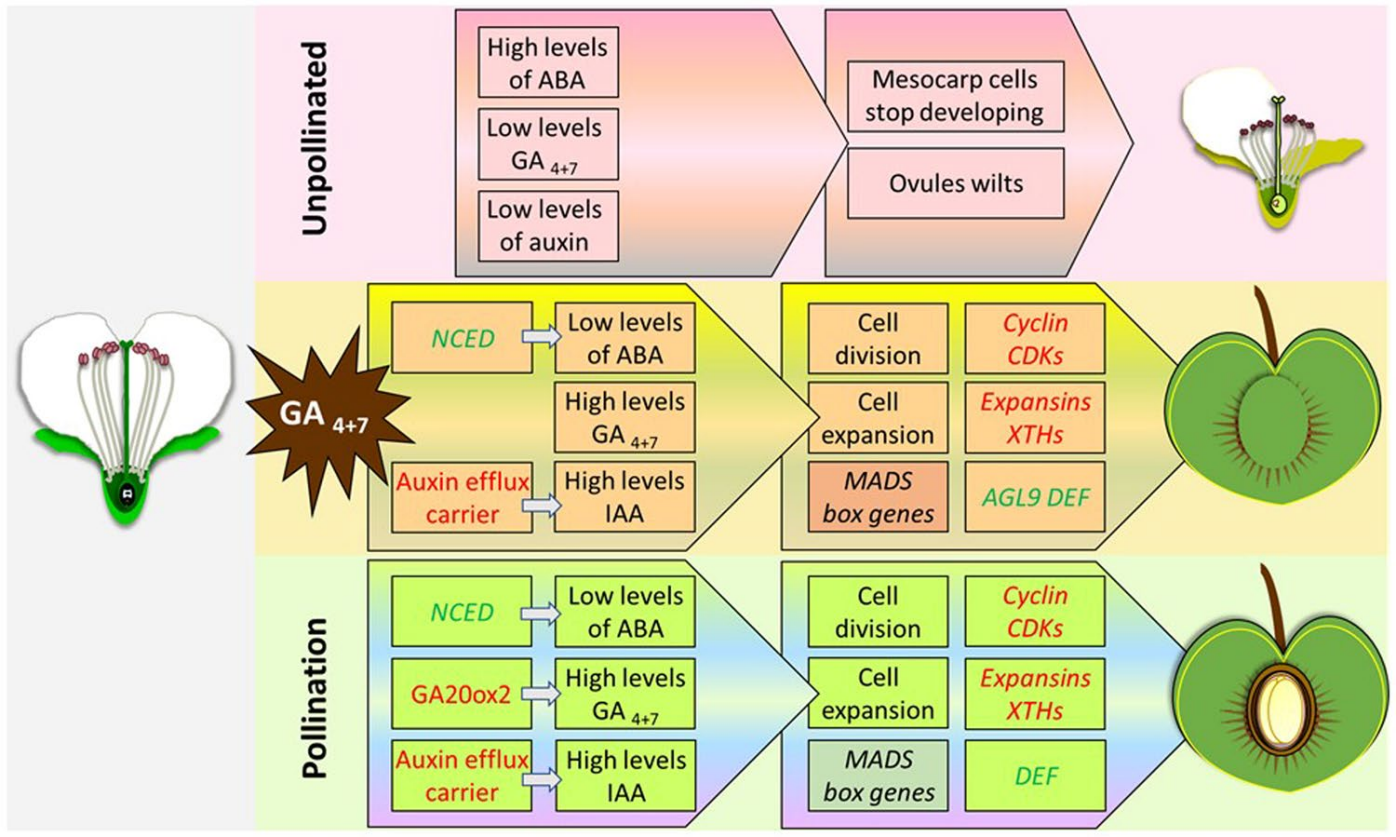
Proposed model of the crosstalk between auxin and GA signalling pathways during fruit development. In this model, three different scenarios are represented: unpollinated, GA_4+7_-treated, and following pollination. Red font represents genes up-regulated and green font genes down-regulated

GAs have also been shown to promote the transcriptional activation of many other genes that are involved in fruit development. For example, transcriptome analysis of the “Dangshansuli” pear treated with GA_4+7_, which induces parthenocarpic fruits, showed that 31 cyclin and 2 CDK genes were up-regulated at 3 DAT, while in the pollinated control, this occurred at 9 DAT. Also, genes encoding expansins, enzymes involved in the modification of the cell wall, have been shown be up-regulated in both the GA_4+7_-treated and pollinated ovaries. Briefly, 103 cell wall-related DEGs have been identified between unpollinated and GA_4+7_-treated ovaries and 72 DEGs between unpollinated and pollinated ones at 3 DAT. Moreover, the number of differentially expressed transcription factor genes with similar expression patterns between GA_4+7_ and pollinated ovaries increases with time (32 at 3DAT, 139 at 9 DAT and 329 at 14 DAT). These transcription factors belong mostly to the bHLH, MYB, NAC, and WRKY families. The treatment with GA also influences the other hormonal pathways, since the treated ovaries showed a small but significant increase of IAA at 3 and 14 DAT, and a significant drop of ABA. Indeed, *PbARF5, PbARF6, PbARF18, PbARF19-like*, and *Pb9-cis-epoxycarotenoid dioxygenase* (NCED), which encode key enzymes in the steps for the synthesis of ABA (Schwartz et al. 1997, 2003; Simkin 2021), have been found to be down-regulated both in the GA_4+7_-treated and pollinated ovaries. Therefore, the model proposed by Liu and collaborators suggests that GA_4+7_ mimics the effect that pollination has in changing the hormones levels, causing a downstream change of expression of gene involved in different aspects of the transition from ovary to fruit (Liu et al. 2018) (Fig. 3).

### Auxins can act as an alternative signal replacing pollination and fertilization

Auxins not only plays a central role in embryogenesis, but also during the development of the other two seed structures, the endosperm and the seed coat. This class of compounds seems to be the driving force behind the development of all the seed components, and to be involved in their growth synchronization. The main type of auxin in higher plants is indole-3-acetic acid (IAA) the biosynthesis of which starts from tryptophan through a process that comprises two steps and is well conserved among higher plants (Mano and Nemoto 2012; Zhao 2014).

It has been proposed that auxin(s) can act as an alternative signal replacing pollination and fertilization to initiate the fruit growth (Serrani et al. 2008)*.* Indeed, exogenous application of auxin to unfertilized ovules or ectopic production in the central cell is enough to activate mitosis in this cell and starts endosperm development (Figueiredo et al. 2015). Interestingly, the “Dangshansuli” pear emasculated pistils treated with the synthetic auxin 2,4-D have been shown to produce parthenocarpic fruits, which are significantly smaller than the seeded fruits. Furthermore, ovaries treated simultaneously with 2,4-D and Paclobutrazol (PBZ) show a significant reduction of cortex thickness, cells’ division, and cell expansion. PBZ is a triazole plant growth regulator that inhibits cell elongation and internode extension by inhibiting gibberellin biosynthesis (Davis et al. 1991). Moreover, in the ovaries treated with 2,4-D, two pear cyclin-dependent genes, *Pbcyclin-dependent kinase B2-2* and, *Pbcyclin-dependent kinase B2-2-like,* and one expansin gene, *Pbexpansine-A10*, were up-regulated. On the contrary, they demonstrated down-regulation when co-application with PZB occurred.

### Auxin acts upstream of gibberellin

**Fig. 4:**
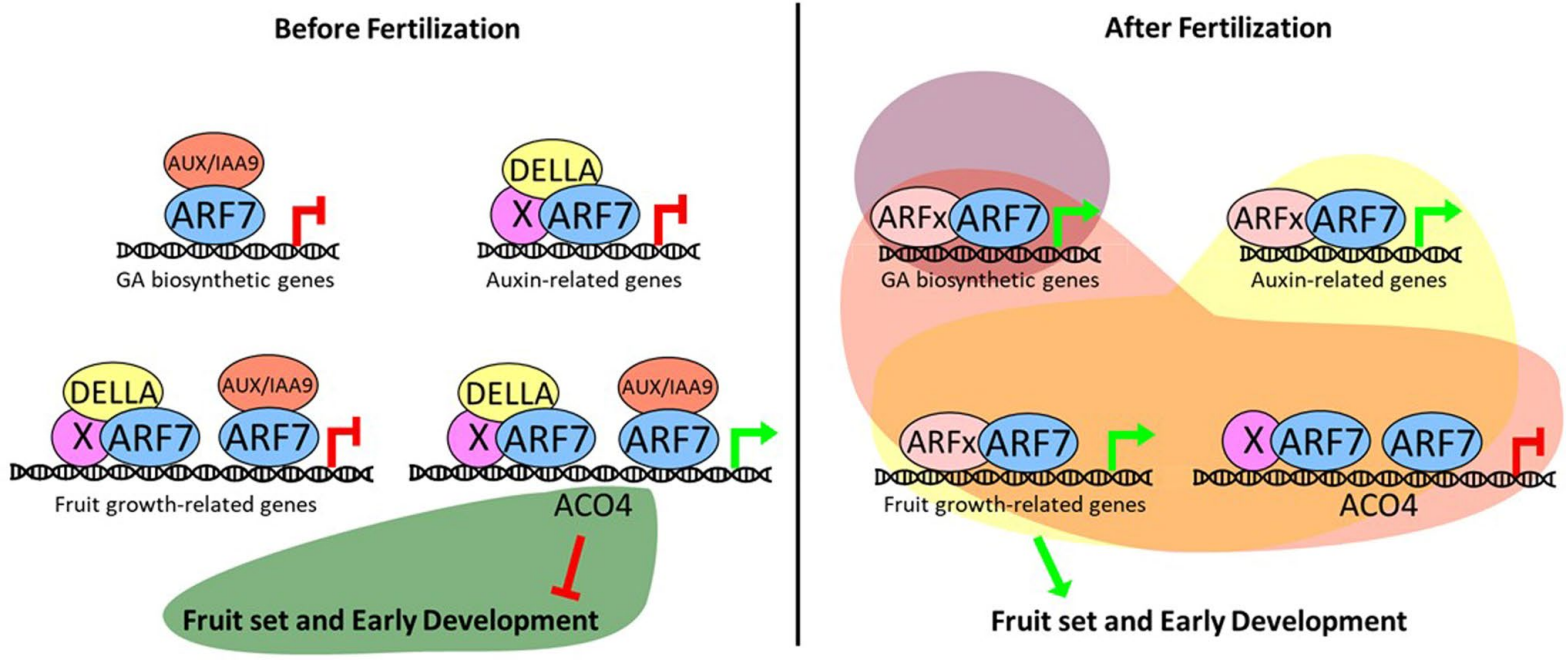
Proposed model of the crosstalk between auxin and GA signalling pathways during tomato fruit development. Before fertilization, SlARF7 (Auxin Response Factor 7) interacts with SlDELLA and SlAUX/IAA9, repressing the transcription of GA biosynthetic genes and the auxin-related genes. They also activate the transcription of ACO4, enhancing ethylene (green area) levels in the ovary which stays frozen. After fertilization, auxin signal (purple circle) comes from the fertilized ovule, promoting the degradation of SlAUX/IAA9. Gibberellins (yellow area) are synthetised and promote the degradation of SlDELLA and a further accumulation of auxin (pink area). SlARF7 can interact with other SlARF proteins and promote fruit set

Auxin seems to act upstream of GA in the induction of fruit set, since auxin can trigger GA biosynthesis but not vice versa (Dorcey et al. 2009). 2,4-D mostly induces the production of bioactive GA_4_ (fourfold increase) but not GA_3_, in the treated ovaries and a decrease when PBZ was also applied. The final evidence consists of the upregulation of *PbGA20ox2-like* and *PbGA3ox-1*, involved in the production of GA_4_, and the down-regulation of *PbGA2ox1-like* and *PbGA2ox2-like*, which converts the GA_4_ precursors into an inactive form (Cong et al. 2019). Furthermore, in tomato, auxin-induced parthenocarpic fruit development is mediated partially by GA, since co-application of auxin and PBZ causes a significant reduction in parthenocarpy (Serrani et al. 2008). Moreover, bioactive GAs levels are high in the auxin-induced and entire parthenocarpic fruits because of the increased expression of GA biosynthetic genes and the repression of GA catabolism genes (Mignolli et al. 2014; Serrani et al. 2008). However, while IAA application promotes the formation of a parthenocarpic fruit by increasing the pericarp cell layers and enlarging the placenta, GA-treated fruits have fewer cell layers, but cells are larger. Parthenocarpic fruits induced by simultaneous application of IAA and GA display a final size and cellular structure that are similar to the seeded fruit. Through different types of experiments, such as Yeast two Hybrid (Y2H), Y3H, and co-immunoprecipitation, it has been shown that SlARF7, which is an activator of ARF, interacts both with SlIAA and SlDELLA, through different regions (Hu et al. 2018) (Fig. 4).

In addition, ARF7 can form homodimers and heterodimers, but while the homodimerization is not affected by the interactions with SlDELLA or SlIAA9, the heterodimerization is slightly affected. A luciferase assay demonstrated that while *GA20ox1, GA3ox1*, and *GH3.2*, which encode an auxin-amino acid conjugating enzyme that converts auxin to an inactive form, are repressed by SlARF7–SLIAA9 complex, they are induced by the SlDELLA-SlARF7 complex. Interestingly, SlDELLA seems to antagonize the SlARF7–SLIAA9 repressive effect on the target genes, such as *Expansin5 (EXP5)* and *ACC oxidase4 (ACO4),* which is an ethylene biosynthetic gene. As mentioned above, simultaneous application of auxin and GAs produces the formation of a fruit with a structure similar to the seeded fruit, and this has been observed also in the *e pro* and *SlARF7i pro* double mutants, where both the pathways are affected. This can be explained by the fact that in tomato, the crosstalk between GAs and IAA, during the fruit set, occurs through DELLA, ARFs, and IAA9 (Hu et al. 2018). These data and those highlighted by Sharif et al. (Sharif et al. 2022) demonstrate that synergistic and antagonistic crosstalk between hormones is essential for determining the fate of fruit set.

### Genetic control of seed development

Seed development is a very elaborate process where three different components develop, each fundamental for reproductive success. Although seed development still has many unknown elements that have to be understood, some important discoveries have been made, especially in *Arabidopsis thaliana*, and one aspect that is clear is that not only is there synchronization of growth of the embryo, endosperm, and seed coat, but also it seems that there is a crosstalk between them, a transfer of signals and information that regulates and coordinates this elaborate process. The *TRYPTOPHAN AMINOTRANSFERASE OF ARABIDOPSIS (TAA/TAR)* gene family encodes enzymes that conduct auxin biosynthesis during the first step, while the *YUCCA (YUC)* family works during the second step. There are also alternative biosynthetic pathways, but the *TAA/TAR-YUCCA* one is the major pathway (Mashiguchi et al. 2011). Mutations in either of the two steps of auxin biosynthesis led to aberrant embryo phenotypes: the *yuc1 yuc4 yuc10* quadruple mutant and the *taa1 tar1 tar2* triple mutant have similar phenotypes, with abnormal division of the hypophysis, rootless seedlings, very short or no hypocotyl and the majority of them has only one cotyledon (Cheng et al. 2007; Stepanova et al. 2008). Notably, mutants in auxin biosynthesis show aberrant embryo phenotypes of the same type as those present in mutants in which the transport of auxin is affected. More precisely, loss of PIN functionality leads to severe effects on embryo development, i.e., the level of aberration increases with the higher loss of PINs. According to their phosphorylation state, PIN proteins change their intracellular localization. If they are phosphorylated, they are targeted to the apical part of the plasma membrane, while if dephosphorylated, they are targeted to the basal part of the plasma membrane. The phosphorylation and dephosphorylation is effected by PID and PROTEIN PHOSPHATASE 2A (PP2A), respectively, which are serine–threonine protein kinases; *pid and pp2a* mutants exhibit similar phenotypes to the one reported for the pin mutants, where the auxin distribution is altered (Bennett et al. 1995; Benjamins et al. 2001; Friml et al. 2004; Michniewicz et al. 2007). In addition, the correct perception of auxin, together with AUX/IAA degradation, is fundamental as well during embryogenesis, since mutants display the same phenotypes as those observed when either biosynthesis or transport are impaired. Mutation of the F-box protein, TIR1, which is involved, upon auxin binding, in the degradation of AUX/IAA proteins, is not enough to affect embryo development, since redundancy has been observed with the closely related AUXIN SIGNALLING F-BOX PROTEIN 1 (AFB1), AFB2, and AFB3 (Dharmasiri et al. 2005a, b; Kepinski and Leyser 2005; Tan et al. 2007). Indeed, the quadruple mutant shows defects in embryogenesis, because it often fails to produce the hypocotyl and the root, and has only one cotyledon (Parry et al. 2009). AUXIN RESPONSE FACTOR (ARF) proteins are fundamental players in auxin signalling, and for this reason, there is a quite high degree of redundancy, so it is unlikely that a mutation in only one of these genes leads to a strong phenotype. The only factor that does not follow this is *MONOPTEROS/ARF5 (MP/ARF5)* whose mutant is rootless, because it exhibits an aberrant division pattern in the hypophysis (Berleth and Jurgens 1993; Hardtke and Berleth 1998; Weijers et al. 2006). Moreover, most of the defects observed and reported in mutants where auxin biosynthesis, transport, or signalling is affected can be explained by the alteration in the activity of MP/ARF5. Indeed, some of the targets of MP are essential transcription factors involved in embryogenesis; these include the *WUSCHEL RELATED HOMEOBOX 9 (WOX9)* transcription factor gene, which is differentially expressed during the early embryo development. Mutations in *MP*, *WOX2*, *WOX8*, and *WOX9* have synergistic effects, meaning that both regulatory pathways converge in controlling the same embryo patterning processes (Haecker et al. 2004); *mp*, *pin1*, and *pid* mutants all fail to demonstrate separate cotyledons and to establish the bilateral symmetry of the cotyledons. Two NAC transcription factor genes, *CUP SHAPED COTYLEDON1 (CUC1)* and *CUC2*, are responsible for the formation of the boundaries between different developing areas and work redundantly to regulate the initial formation of the SAM and the separation of the cotyledons, together with *SHOOT MERISTEMLESS (STM).* While *CUC1* and *CUC2* are restricted to the cotyledon margins, *STM* is localized only in the SAM (Aida et al. 1997, 1999). *MP*, *PIN1*, and *PID* have been observed to be required for the activation of *CUC2* in the boundaries and the repression of *CUC1* in the cotyledons (Aida et al. 2002; Furutani et al. 2004). Mutation of *CUC1* partially restores the correct cotyledon development in the double mutant *pin1pid*, meaning that probably the defects observed are partially caused by an ectopic expression of *CUC1* in the cotyledons (Furutani et al. 2004). However, it is still unclear if MP directly regulates the expression of CUC genes or just promotes *PIN1* expression and this has a downstream effect on *CUC1* and *CUC2*.

*YUC10* and *TAR1* have been demonstrated to be imprinted in the endosperm and paternally expressed. This epigenetic regulatory mechanism is conserved among distantly related species, such as *Oryza sativa*, *Capsella. rubella*, and *A. lyrata* (Du et al. 2014; Hatorangan et al. 2016; Klosinska et al. 2016; Luo et al. 2011). Another example is strawberry, where the homologues of *YUC10* and *TAR1* are specifically expressed in the endosperm, although their imprinting has not yet been determined (Kang et al. 2013). An interesting hypothesis is that there is a sort of auxin transport from the endosperm to the embryo, and since *YUC10* and *YUC11* are specifically expressed in the endosperm, this could explain the defects observed in the embryos of those mutants (Figueiredo et al. 2015; Figueiredo and Köhler 2018; Robert et al. 2013). To support this hypothesis, it is known that the auxin importer gene *LAX1* is specifically expressed in the tip of the cotyledons, but to date, the import of auxin from the endosperm has not yet been demonstrated (Robert et al. 2015). In Arabidopsis, in particular, it has been demonstrated that if auxin biosynthesis or signalling is altered, there is a dramatic effect on the endosperm proliferation rate. Another example of the crucial role of auxin in endosperm development can be found in the woody plant *Jatropha curca*, and in particular, *JcARF9* has been proven to activate cell cycle regulators in the endosperm, such as *JcCKA1*, *JcCYCD2*, and *JcCYCD5* (Sun et al. 2017). Endosperm development is also highly sensitive to auxin in both raspberry and blackberry seeds (Jennings et al. 1967; Jennings 1971). However, although this crucial role of auxin during endosperm development has been observed, very little is known about its dynamics and transport during the process.

Seed coat development has been proven to be triggered only after fertilization of the central cell and not of the egg cell, meaning that the endosperm is involved in sending signals to the ovule integuments to initiate their conversion to seed coat (Roszak and Köhler 2011; Weijers et al. 2003). However, no cytoplasmic connections have been found between the endosperm and the seed coat, and thus, the communication between these two structures has to rely on small molecules such as peptides and hormones that can cross the plasma membrane (Figueiredo and Köhler 2016; Ingram 2010). Further experimental evidence that auxin produced in the endosperm affects seed coat development has been found in plants where auxin biosynthesis is down-regulated in the endosperm, resuting in seed coat development being significantly altered. Both the transport of auxin from the endosperm to the embryo, and the transport from the triploid tissue to the seed coat remain to be investigated. MADS-box transcription factor *AGAMOUS-LIKE62 (AGL62)* mutant has been shown to lack the ability to export auxin to the seed coat, and as a consequence, to form it, although the reason why is still unclear. It also appears that epigenetics has a role in the initiation of the seed coat development, blocking it in the unfertilized ovule, and that auxin plays a role in the regulation of this epigenetic mechanism (Figueiredo and Köhler 2016; Roszak and Köhler 2011). Epigenetics is also involved in endosperm development, since a large group of the polycomb repressive complex (PcG) proteins have been identified in the endosperm (Pien and Grossniklaus 2007).

Furthermore, auxin is not the only hormone that has a role during seed development. Gibberellins (GAs) are tetracyclic diterpenoids and they are required for the correct development of the seed, since the GA-deficient mutants display an altered seed development and a significant level of abortions. In tomato, the GA-deficient mutant *ga-1* displays altered development, not only of the seed, but also of the flower and the fruit (Groot et al. 1987), while in pea, the *lh* mutant, which has a significant reduction in the synthesis of GA, has an clear decrease in the seed weight and reduction in seed number in each pod (Swain et al. 1993, 1997). Another example comes from a study involving the constitutive overexpression of the pea *GA2 oxidase2 (GA2ox2)* gene, which encodes for an enzyme that irreversibly converts active GAs and their precursors into the inactive forms. In Arabidopsis, this causes seed abortion at different stages of the developmental process, since only 3% of the viable seeds carry the *GA2ox2* transgene. Thus, the effect of the over expression of *GA2ox2* reduces the seed number, due to seed abortion, and the fruit size. However, it has not been possible to measure the GA levels in the aborted seeds because of their small size, so although it is likely that the abortion of the seeds was caused by a low level of active GAs, it has not been demonstrated in this study (Singh et al. 2002). The target of the GA signalling pathway is the *Gibberellic Acid Stimulated Arabidopsis (GASA)* gene family whose expression is activated and stimulated in response to GAs (Herzog et al. 1995). *GASA4*, for example, is activated in the shoot apex, in the developing flowers and embryos, and its overexpression leads to a significant increase in seed size and seed weight, while the gasa4 mutant has smaller seeds (Roxrud et al. 2007). Gibberellins act downstream of auxin, since exogenous application of the latter to unfertilized ovules is enough to activate the transcription of the GA biosynthetic genes (Dorcey et al. 2009). Finally, a predominant role of cytokinins has been observed during the early endosperm development, influencing the final dimension of the seed (Day et al. 2008). Arabidopsis histidine kinases (AHKs) are used by the plant cells as cytokinin receptors, and they target the Arabidopsis histidine phosphotransfer proteins (AHPs), which are involved in cytokinin signal transduction. Both *ahk2ahk3ahk4* and *ahp2ahp3ahp5* triple mutants produce seeds that are twice the size of those in the wild type (Hutchison et al. 2006; Müller and Sheen 2007; Riefler et al. 2006).

As mentioned above, MADS-box transcription factor genes are also involved in ovule seed development, and among these *SEEDSTICK* (*STK*)*, SHATTERPROOF 1* (*SHP1*) and *SHP2* are included. Indeed, the *stk shp1 shp2* triple mutant has a visible funiculus, but the integuments are converted to carpel-like structures, showing that these three genes together are indispensable for ovule identity determination (Brambilla et al. 2007; Pinyopich et al. 2003). Moreover, STK has been shown to work in association with the MADS-box transcription factor ARABIDOPSIS B_sister_ (ABS) in the development of the endothelium, which is the innermost layer of the seed coat, directly in contact with the endosperm. The *stk abs* double mutant, which lacks the endothelium presence in the seed coat, shows a great reduction of seed production due to abortions during both ovule and seed development (Mizzotti et al. 2012). Moreover, STK is responsible for the correct formation of the cell wall in the seed coat, since it directly regulates factors that take part in the biosynthesis of the cellulose–pectin matrix of the cell wall (Ezquer et al. 2016). In addition, the *stk* mutant not only shows cells in the seed coat with an altered morphology, but also an altered accumulation of proanthocyanidins. RNA-seq and in-situ hybridization and ChIP data have shown that STK binds the promoter of *BANYULS/ANTHOCYANIDIN REDUCTASE (BAN/ANR),* which is involved in the proanthocyanidins biosynthetic pathway. Therefore, STK is also involved in the control of secondary metabolism in the seed coat (Mizzotti et al. 2014). Finally, STK seems to be essential for correct seed development, and its mutation can lead to the production of a stenospermocarpic fruit.

### Seedless fruit has been identified in multiple species: can this information provide a route to the development of a seedless cherry?

#### Parthenocarpy and stenospermocarpy in fruiting plants

In some horticultural crops, fruit set and development can occur without the fertilization of the ovules in a process known as parthenocarpy (from Greek “virgin fruit”) (Picarella and Mazzucato 2019; Sharif et al. 2022). Natural parthenocarpy can be obligated in sterile species, such as banana and pineapple, or facultative as in some tomato mutants.

Parthenocarpy has been reported in 96 Angiosperm taxa (Gustafson 1942; Picarella and Mazzucato 2019). The group that presents the highest number of parthenocarpic fruits is the Rosidae (49.8%), where the Rosaceae contribute with six species (the second bigger contribution after the Anacardiaceae and the Rutaceae with eight species). Interestingly, about a half of the parthenocarpic species are trees and between the monospermic fruit-type species, the ones that develop a drupe-type fruit make up the majority. In particular, *Prunus persica* (L.) Batsch and *Prunus cerasifera* Ehrh are the two examples of woody plants with a drupe-type fruit that belong to the Rosaceae, in which parthenocarpy had been reported (Gustafson 1939, 1942; Picarella and Mazzucato 2019). Parthenocarpy is an important agricultural trait that can mitigate poor fruit setting caused by unfavorable pollination conditions (Gou et al. 2022). Similarly, seedless fruit can be formed by a preocess termed stenospermocarpy, which is the formation of fruit with spontaneous or induced abortion of fertilized seeds (watermelon and grapes). However, this process, unlike parthenocarpy, requires pollination.

#### Genetic insights of seedlessness

The MADS-box transcription factors involved in the identity determination of the floral whorls, according to the ABCDE model (Theißen 2001), have been observed to be involved in the formation of a seedless fruit. Indeed, the apple mutant Rae Ime has been reported to be parthenocarpic if not pollinated, while if it is pollinated, it produces seeds with smaller embryos. Its flowers have no petals and stamens, while an apple wild-type flower has five petals and between 9 and 20 stamens, Rae Ime also posesses two whorls of five sepals and an increased number, up to 15, styles and carpels, while the wild-type flower typically has five (Pratt 1988; Yao et al. 2001).

There are other apple mutants, Spencer Seedless and Wallington Bloomless for example, which produce flowers with a similar phenotype of Rae Ime, and are parthenocarpic. This phenotype is similar to that observed in the Arabidopsis mutants *pi* and *ap3*. *PISTILLATA (PI)* and *APETALA3* (*AP3*) belong to the B-class and are involved in the identity determination of the petals, and stamens, with the A-class and C-class MADS-box transcription factors, respectively (Goto and Meyerowitz 1994; Jack et al. 1992; Theißen and Saedler 2001). It has been observed that the apple gene *MdPI*, which encodes a protein with 64% identity to that encoded by *AtPI* and which has a similar expression pattern compared to Arabidopsis, in Rae Ime has a 9332 bp LRT-retrotransposon insertion that affects the function of this gene. The mutation of *MdPI* has also been found in Spencer Seedless and Wallington Bloomless (Table 1) (Yao et al. 2001). Furthermore, co-suppression of *MdPI* in transgenic apple was also shown to result in parthenocarpy [see Tanaka and Wade (2022) for review].

**Table 1:** Summary of the genes involved in parthenocarpic fruit development and potential targets to induce parthenocarpy in cherry

Target	Pathway	Plant	Phenotype	Reference
Auxin Response Factor 7 (ARF7)	Auxin	Arabidopsis	Parthenocarpy, seedless/pseudoembryos, size, and shape similar to WT	Goetz et al. (2007)
		Tomato	Down-regulation of ARF7 resulted in the formation of parthenocarpic fruit and altered shape. SlARF7 RNAi lines also display a down-regulation of SlARF5 and SlARF8B, suggesting that ARF7 cannot promote parthenocarpy unless ARF5 levels are also reduced	de Jong et al. (2009, 2011); Hu et al. (2018)
Auxin Response Factor 8 (ARF8)	Auxin	Eggplant	Natural parthenocarpic mutant showed that ARF8, is down-regulated in buds compared to wild-type plants. Transgenic RNAi lines of ARF8 exhibited parthenocarpy in unfertilized flowers. ARF8 negatively regulates fruit initiation	Du et al. (2016)
		Arabidopsis	Mutations in ARF8 uncouple fruit initiation from fertilization, resulting in the formation of seedless, parthenocarpic fruit/pseudoembryos, size, and shape similar to wt	Goetz et al. (2007)
		Tomato	Expression of an aberrant ARF8 mutant transcript from Arabidopsis in tomato results in parthenocarpy	Goetz et al. (2007)
Indole-3-Acetic Acid Inducible 9 (IAA/9)	Auxin	Tomato	down-regulation of IAA9 resulted in parthenocarpic fruit and auxin-related alterations in the leaf morphology	Zhang et al. (2007)
			Parthenocarpy, seedless, early fruit growth, normal size, and shape	Wang et al. (2005)
AUCSIA	Auxin	Tomato	Aucsia gene silencing causes parthenocarpic fruit development in tomato. Aucsia silenced tomato plants are characterized by facultative (seedless fruits when flowers are emasculated) and rarely, obligate parthenocarpy (i.e., seedless fruit from pollinated flowers). The facultative parthenocarpic fruits were similar in shape to wild-type fruit	Molesini et al. (2009, 2020)
PIN4	Auxin-transport	Tomato	The mutation of the auxin efflux carrier encoding gene PIN4 in tomato has been reported to trigger the development of parthenocarpic fruit	Mounet et al. (2012)
DELLA	GA	Arabidopsis	Parthenocarpy observed in DELLA mutants is directly attributed to the constitutive activation of GA signalling	Fuentes et al. (2012)
DELLA Pistillata (PI)	GA MADS-Box	Arabidopsis	DELLA mutants have impaired fertilization (seed set)	Dorcey et al. (2009)
		Tomato	Silencing of DELLA induces facultative parthenocarpy, seedless fruit with reduced size, altered morphology	Martí et al. (2007); Livne et al. (2015); Carrera et al. (2012)
		Apple	Abolishing the normal expression of the Pistillata gene in apple confers parthenocarpic fruit development	Yao et al. (2001)
Sepallata (SEP)	MADS-Box	Tomato	Antisense and co-suppression of Sepallata in the transgenic ovary developed into parthenocarpic fruit without pollination. The transgenic fruit are bigger than the wild type, although it remains green for a longer before starting the maturation process	Ampomah-Dwamena et al. (2002)
SEEDSTICK (STK)	MADS-Box	Arabidopsis	Controls structural and mechanical properties of the Arabidopsis seed coat	Ezquer et al. (2016)
AGAMOUS like-6 (AGL6)	MADS-Box	Tomato	Seedless fruits are of normal weight and shape. Down-regulation in natural mutant causes parthenocarpy and low seed	Klap et al. (2017); Takisawa et al. (2018)
AGAMOUS like-11 (AGL11)	MADS-Box	Tomato	Gene silencing of AGL11 in tomato produces seedless fruits. Seedlessness is proportional to transcript accumulation levels	Ocarez and Mejía (2016)
		Tomato	Gene silencing of both AGL11 and a second MADS-Box gene, MBP3, produces all flesh seedless fruit exhibit enhanced firmness and improved post-harvest storage	Huang et al. (2021)
		Grape	Seed abortion caused by a single amino acid substitution in VviAGL11 is the major cause of seedlessness	Royo et al. (2018)
		Grape	Dominant mutation in VviAGL11, homologue of STK, are seedless Seeded fruit is bigger than seedless fruit due to higher expression of VviAGL11	Mejía et al. (2011)

*SEPALLATA* (*SEP*) genes encode for E-class MADS-box transcription factors, which are necessary for the function of all the other classes. Indeed, the *sep1sep2sep3* triple mutant displays an indeterminate flower growth where all the whorls are composed of sepals (Ditta et al. 2004; Pelaz et al. 2000; Theißen 2001).

Tomato MADS-box 29 (*TM29*) is a single copy gene and has been shown to be the *SEPALLATA* homolog in tomato, since the encoded protein shares a 68%, 63%, and 58% amino acid sequence identity with AtSEP1, AtSEP2, and AtSEP3, respectively, and also, the expression pattern is conserved. In addition, *TM29* has been found in the vegetative and inflorescence meristems, so it seems to have an additional function compared to SEP1. Down-regulation of *TM29* through RNAi in tomato leads the production of a mutant with some flower defects, such as green and sterile anthers, and a pistil that develops a parthenocarpic fruit without pollination and fertilization (Table 1). The transgenic fruit is bigger than the wild type, although they remains green for a longer time before starting the maturation process. Moreover, ectopic shoot formation has been observed in the transgenic fruit, which becomes swollen and misshapen. The shoot produces in turn a new fruit from which a new ectopic shoot arises, and this cycle can be repeated three or four times (Ampomah-Dwamena et al. 2002). This severe phenotype can be explained by the fact the *SEPALLATA* genes are very basal factors (E-class) that work in the determination of all the whorls.

**Fig. 5:**
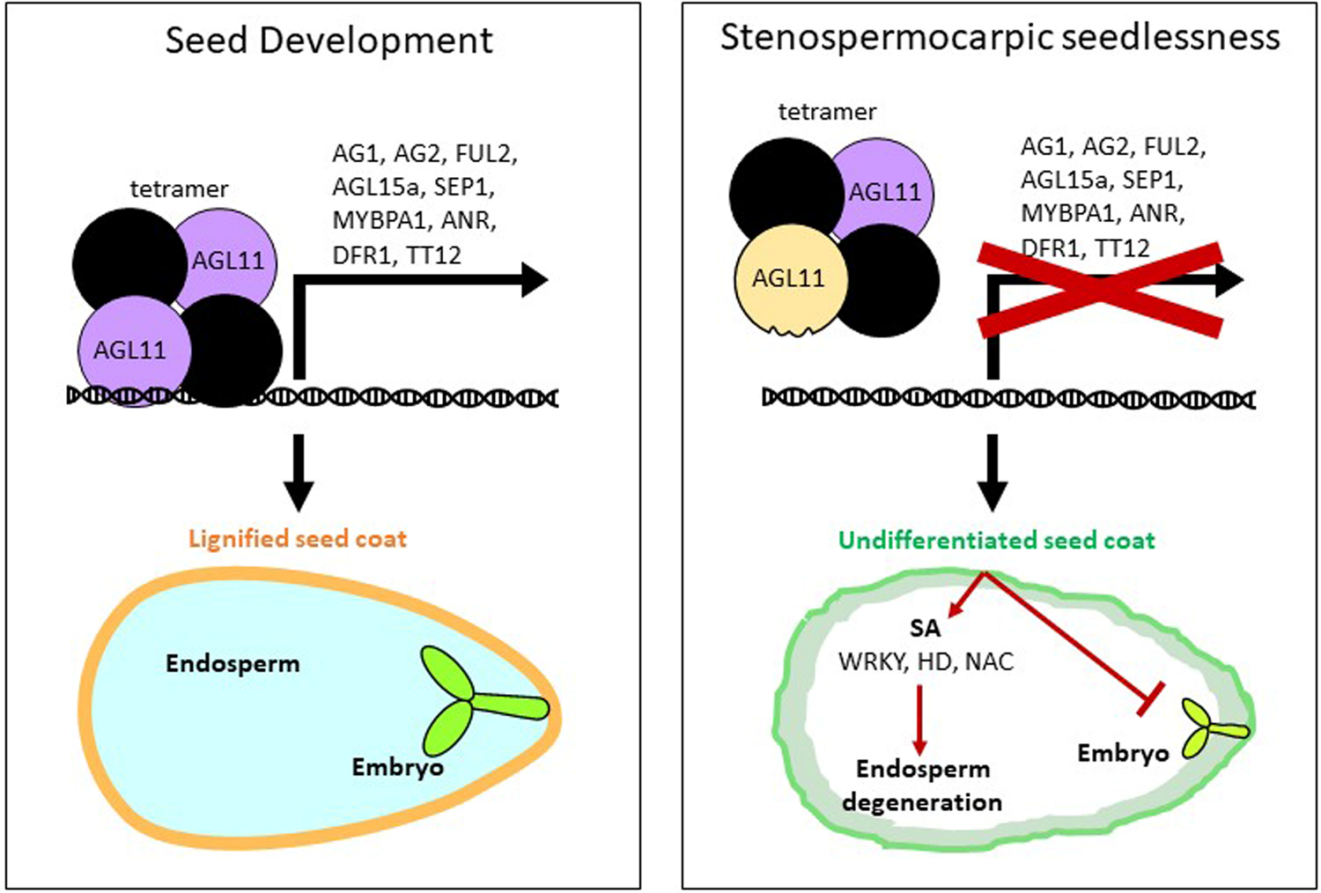
Model of seedlessness proposed by Royo et al. (Royo et al. 2018) to explain the seedless phenotype in grape. The panel on the left shows the initiation of seed morphogenesis that occurs under normal development. The wild-type VviAGL11 (AGAMOUS-LIKE11) protein complex (shown in purple) either directly or indirectly initiates the expression of genes involved in seed coat sclerification, permitting embryo development. The panel on the right shows a heterozygous individual with a dominant phenotype due to a single missense mutation in the VviAGL11 protein (Arg197 to Leu197; shown in yellow). This mutation prevents the assembly of the multiprotein complex halting seed coat differentiation, leading to degeneration of the embryo and endosperm

The opposite scenario can be observed in tomato and grapevine mutants of *TOMATO AGAMOUS-LIKE 11 (AGL11/TAG11)* and *Vitis vinifera AGAMOUS-LIKE 11 (VviAGL11)* respectively (Table 1). TAGL11 is the STK homolog in tomato, it shares 81% similarity at the nucleotide level with the Arabidopsis gene, and it controls fruit and seed development, being expressed in the inner integuments of the ovule. *TAG11* has been silenced using an antisense construct and the lines produced do not exhibit any significant difference in fruit number, date of maturity, final ripening colour, and fruit size. Most of these lines are seedless, and some have few seeds, smaller seeds, or ovule traces. The number of seeds is positively correlated with the expression level of *TAG11* in these lines (Ocarez and Mejía 2016). Further work by Huang et al (2021) used crispr/cas9 to silence *AGL11* and a second MADS-Box gene, *MBP3* in the same lines. This work resulted in the production of all-flesh, seedless transgenic lines with enhanced firmness and improved post-harvest storage; however, average fruit size was severely reduced. Similar results have been reported in pepper, with parthenocarpic fruit reported to be 30% smaller than seeded fruit (Heuvelink and Körner 2001). These data suggest that multi-target approaches may be necessary to achieve seedless cherry and maintain fruit size. *VviAGL11* is the homolog of *STK* in grapevine and it starts to be highly expressed when the seed begins to develop, immediately after berry set (Mejía et al. 2011). Comparing different seeded and seedless genotypes of grapevine has shown that the seeded ones have bigger fruit and a higher expression of *VviAGL11* than the seedless ones. Seedless Ruby is a stenospermocarpic mutant that has a dominant mutation in *VviAGL11*, since the heterozygous plants are seedless. The dominant phenotype is due to a single missense mutation (Arg197 to Leu197), which does not allow the formation of the correct transcriptional complex that is involved in activating all the genes necessary for the normal seed coat development and lignification, which, as mentioned in the previous paragraphs, is crucial for the correct endosperm and embryo development (Ocarez and Mejía 2016; Royo et al. 2018) (Fig. 5).

Two members of the Auxin Response Factor (ARF) family, *ARF7* and *ARF8*, have recently been implicated in fruit initiation (Table 1). The transcript levels of both genes are highly expressed in non-pollinated flowers and are down-regulated after pollination. Down-regulation of *ARF7* in tomato resulted in the formation of parthenocarpic fruit and a significant reduction in the accumulation of GAs compared to seeding fruit (de Jong et al. 2009, 2011). Hu et al. (2018) reported that *SlARF7* RNAi lines also display a down-regulation of SlARF5 and SlARF8B. These results clearly demonstrate that ARF7 acts to modulate both auxin and gibberellin during fruit set and mediates crosstalk between auxin and gibberellin signalling during tomato fruit development; however, the co-down-regulation of *SlARF5* suggests that *SlARF7* cannot promote parthenocarpy unless *SlARF5* levels are also reduced.

Similar results have been found with *AUXIN RESPONSE FACTOR8 (ARF8)* in tomato, eggplant, and Arabidopsis. In Arabidopsis, *ARF8* mutants display parthenocarpic siliques and the expression of the Arabidopsis mutant allele in tomato results in the production of seedless fruits (Goetz et al. 2006, 2007; Du et al. 2016). The introduction of the mutant *ARF8* allele into Arabidopsis did not result in the suppression of the endogenous *ARF8* transcript; however, the aberrant transcripts compromised the function of endogenous *ARF8* (Goetz et al. 2007). It has been proposed that in both Arabidopsis and tomato, ARF proteins can bind Aux/IAA proteins to form a protein complex that inhibits or activates auxin responsive genes (Hardtke et al. 2004; Tatematsu et al. 2004; Ulmasov et al. 1999). The accumulation of the mutant transcript is thought to destabilize the formation and/or function of the ARF-IAA complex, permitting fruit set in the absence of pollination (Goetz et al. 2006; Swain and Koltunow 2006). Furthermore, the down-regulation of *Indole-3-acetic acid inducible 9* (*IAA9)* (Table 1), the second member of this complex, using an antisense approach in tomato, resulted in parthenocarpic fruit development and auxin-related alterations in leaf morphology (Wang et al. 2005). Kim et al (2020) reported that the silencing of *AUX/IAA9* in tomato mimics an increase in auxin levels in the unpollinated ovaries and promotes the expression of auxin and gibberellins biosynthetic genes, which are activated during the fruit set. Auxin and GA crosstalk has been better investigated by Hu et al. (2018). Indeed, the tomato *entire* (SlIAA9 loss of function), *SlARF7* RNAi, and *procera* (SlDELLA loss of function) mutants show strong parthenocarpy.

#### June drop

Fruit trees produce a large quantity of flowers, more than are necessary to produce a healthy crop of fruit. In the case of pears and apples, the trees thin themselves, dropping small fruit so as to not overburden the plant, and thereby reducing the chance that branches will break under excessive weight. This process, often referred to as ‘June drop’ or ‘cherry run-off’, begins in late June and continues until mid-July. If just one in 20 flowers produces a fruit, the tree is carrying a healthy crop. These remaining fruits develop normally, becoming bigger at the expense of the fruitlets lost earlier. However, cherries, and other stone fruit, often do not need thinning and do not drop excess flowers requiring extra manual resources to protect crop yield. However, in some years, sweet cherry trees lose a significant proportion of their fruit before ripening. This loss varies from year to year and, in some seasons, can result in a total loss of the crop. In the UK in 2000, for example, cherry trees lost as much as 90% of the fruit set before harvest.

At the start of fruit drop in cherry, when fruits with aborted seeds are abscising, there is significantly more ethylene in the pedicels than in the flesh of the fruits. In both sweet and tart cherry, ethylene was shown to be elevated (fivefold) in fruit flesh and pedicles of abscising fruit compared to adhering fruits (Blanpied 1972). Furthermore, since the application of endogenous ethylene promote the abscission of sweet and tart cherries (Edgerton and Hatch 1969), the combined data suggest that ethylene may play a role in early fruit drop. Furthermore, polar auxin transport inhibitors increased fruit abscission by 30%, demonstrating that maintaining auxin (indole-3-acetic acid, IAA) transport and concentrations in the abscission zone plays a role in fruit retention (Blanusa et al. 2005; Guinn and Brummett 1987). It has been reported that IAA delays abscission by lowering the cells sensitivity to ethylene (Sexton et al. 1985), and yet also stimulates ethylene production, while ethylene is a potent inhibitor of auxin transport (An et al. 2020). This interplay between these two hormones demonstrates the extreme complexity between the regulation of fruit set and fruit abscission (for review see (An et al. 2020; Taylor and Whitelaw 2001). As previously noted, during fruit development, communication between the seed/embryo and the fruit, necessary for the developmental synchronization during fruit formation, involves phytohormones (auxin and gibberellins) produced either in the developing embryo, endosperm, or in the seed coat (Figueiredo and Köhler 2016; Gillaspy et al. 1993; Ingram 2010; Varoquaux et al. 2000).

The complex inter-hormonal signalling mechanism that identifies an aborting seed to the tree and results in fruit abscission is poorly understood; however, it is probable that either a signal travels from the aborting seed to the tree to induce fruit abscission or there is a loss of signal from the seed coat, embryo, or endosperm, that would normally be present during successful seed development.

### Options for the generation of seedless cherry and intellectual property rights in the domain of seedless fruit

Seed, stone, and fruit development is a tightly controlled process involving key phytohormones, such as gibberellins, auxins, and environmental cues. The absence of the seeds/stone would be advantageous for producers, reducing processing costs and increasing profits and for the consumer. The presence of the stone and seed makes it both inconvenient to eat ‘on the move’ and potentially dangerous for children. Removal of this stone through genetic means could be potentially transformative for the cherry industry and facilitate rapid market expansion in the UK and beyond. Furthermore, parthenocarpic fruits are firmer and fleshier than pollinated fruits due to the absence of the seeds (Gou et al. 2022; Varoquaux et al. 2000).

Exogenous application of plant hormones has been shown to result in the development of parthenocarpic fruit; however, large-scale introduction of such treatments would prove costly to implement; thus, genetic engineering and genome editing strategies aimed at altering hormone biosynthesis or signalling to obtain parthenocarpic fruits seems a preferable approach. This review highlights some of the potential gene targets for the generation of seedless fruit. We have identified, in the genome databases, orthologues of many of these genes in cherry (Table 2). Manipulation of the listed genes have been shown to induce, directly or indirectly, the development of seedless fruit in the listed species. The table highlights the action required in cherry to potentially obtain a seedless cherry. Previous results in tomato have shown that the down-regulation of *ARF7* promotes parthenocarpy; however, this induction requires the co-down-regulation of *ARF5* in these plants (Hu et al. 2018). Orthologues of both *ARF7* and *ARF5* have been identified in the Cherry (Table 2). To obtain parthenocarpic cherry fruit, it may be necessary to knock-down both *ARF7* and *ARF5* simultaneously.

**Table 2:** A list of cherry homologs of the genes involved the development and parthenocarpic fruit in tested species

Gene	Species	Ref	Putative cherry orthologue	Action
ARF8	Arabidopsis, Tomato, Eggplant	Goetz et al. (2007; Du et al. (2016)	*Pav_sc0001314.1_g050.1.mk*	Knock-out
ARF7	Tomato	de Jong et al. (2009)	*Pav_sc0000129.1_g1480.1.mk*	Knock-out
ARF5	Tomato	Hu et al. (2018)	*Pav_sc0002080.1_g180.1.mk*	Knock-out
ARF5-putative	Tomato	Hu et al. (2018)	*Pav_sc0011314.1_g020.1.br* *Pav_sc0003104.1_g030.1.br* *Pav_sc0002826.1_g100.1.br* *Pav_sc0001759.1_g010.1.br*	Knock-out
AUX/IAA9	Tomato, Eggplant	Zhang et al. (2007); Chen et al. (2017)	*Pav_sc0002393.1_g030.1.mk* *Pav_sc0002327.1_g560.1.mk*	Knock-out
AUCSIA	Tomato	Molesini et al. (2020)	*Pav_sc0000175.1_g020.1.mk*	Knock-out
PIN4	Tomato	Mounet et al. (2012)	*Pav_sc0000103.1_g670.1.mk*	Knock-out
DELLA	Arabidopsis. Tomato	Martí et al. (Fuentes et al. (2012; Livne et al. (2015); 2007;	*Pav_sc0000464.1_g350.1.mk*	Knock-out
GA20ox	Arabidopsis, Tomato, Citrus	García-Hurtado et al. (2012); Mesejo et al. (2016)	*Pav_sc0000072.1_g640.1.mk*	Up-regulation
SEPETALLA (TM29)	Tomato	Ampomah-Dwamena et al. (2002)	*Pav_sc0000176.1_g060.1.mk* *Pav_sc0000091.1_g150.1.mk* *Pav_sc0000661.1_g410.1.mk* *Pav_sc0001080.1_g900.1.mk*	Knock-out
PISTILLATA	Apple, Grape	Yao et al. (2001); Fernandez et al. (2013)	*Pav_sc0000195.1_g010.1.mk*	Knock-out
HYDRA	Tomato	Rojas-Gracia et al. (2017)	*Pav_sc0000124.1_g160.1.mk* *Pav_sc0002264.1_g290.1.mk*	Knock-out
AGL6	Tomato	Klap et al. (2017); Takisawa et al. (2018)	*Pav_sc0000072.1_g670.1.mk*	Knock-out
AGL11	Grape, Tomato	Ocarez and Mejía (2016); Royo et al. (2018)	*Pav_sc0002136.1_g280.1.mk*	Knock-out

*Cherry* genes were identifed in the Genome Database Rosaceae: https://www.rosaceae.org/species/prunus_avium/genome_v1.0.a1 and AUCSIA and HYDRA were identified by blast of tomato sequences against the cherry genome on EnsemblPlants. Manipulation of the listed genes has been shown to induce, directly or indirectly, the development of parthenocarpic fruit in the listed species

As previously noted, down-regulation of *DELLA* in tomato causes parthenocarpy (Carrera et al. 2012; Martí et al. 2007; Livne et al. 2015). A single copy of *DELLA* has been identified in tomato. Here, we have identified a single copy of *DELLA* in sweet cherry making it an ideal target for generating seedless fruit. In tomato, we should note that the fruit is smaller than wild type. However, in tomato, a mutation in *DELLA* (*procera*) includes elongated internode length, thinner leaves, and reduced lobing of the main leaflets, which could influence fruit development and fruit size. One option in sweet cherry would be to use a targeted approach, down-regulating *DELLA* at early fruit development and in the fruit leaving wild-type levels in the vegetative tissues. The identified orthologue of DELLA gene in the cherry database, *Pav_sc0000464.1_g350.1.mk* (Table 2), is highly expressed in the unpollinated ovaries at anthesis and decreases only after pollination.

Another option, the down-regulation of the *SEPETALLA* gene in tomato resulted in parthenocarpic fruit that is bigger than the wild type (Ampomah-Dwamena et al. 2002), suggesting that this may be a more viable target in sweet cherry; however, in this instance, four copies of the *PISTILLATA* gene have been identified in sweet cherry genome; therefore, identifying the correct target may be more complex.

Over the last few decades, agricultural research has adopted technologies such as genetic engineering and more recently ‘genome editing’ to improve traits in key crops (Aglawe et al. 2018; Georges and Ray 2017; Simkin 2019; Wilson et al. 2019). There have been recent advances in the tools available to carry out this work, including vectors for multiple gene insertion (Engler et al. 2008, 2009, 2014; Exposito-Rodriguez et al. 2017; Marillonnet and Werner 2015) and tissue-specific promoters (Alotaibi et al. 2018, 2019; Kuntz et al. 1998; Mukherjee et al. 2015; Simkin et al. 2006, 2007).

It has previously been demonstrated that the CRISPR-Cas9 genome editing system can be used to breed parthenocarpic tomato plants (Huang et al. 2021). To use site-directed mutagenesis to obtain knock-out mutants of the cherry orthologues, two factors need to be taken into account. First, fruit trees are included in the group of the most reluctant species for in vitro tissue culture. In vitro cherry regeneration and transformation are remarkably challenging, because it has a low success rate and is time-consuming (Vergara et al. 2021). There are, however, a few regeneration and transformation protocols developed for commercially important cherry varieties; these involve the use of leaves, shoots, cotyledons, and epicotyls as initial explants (Bhagwat and Lane 2004; Blando et al. 2007; Canli and Tian 2008; Feeney et al. 2007; Matt and Jehle 2005; Zong et al. 2019; Vergara et al. 2021). Second, the interest is in confirming the phenotype at the flower and fruit level, and sweet cherry has a long juvenile phase (from 4 to 10 years), since it is a woody plant. This obstacle could be overcome by overexpressing a florigen gene such as *FLOWERING LOCUS-T* (*FT*). In fact, in plum (*P. domestica*), the overexpression of the poplar ortholog of *FT1* has significantly reduced the juvenile phase, allowing to fruits set just 1 year after seed germination (Srinivasan et al. 2012).

The development of a commercially viable stoneless and seedless cherry must take into account current intellectual property rights (Table 3) that may affect where and how a new variety can be marketed and whether licensing agreements are required before a products can be commercialised. *PIN4*, for example, a copy of which has been identifed in sweet cherry (Table 2), known to be involved in the development of parthenocarpic tomato fruit (Mounet et al. 2012), is the subject of an active patent until at least 2036 (Van Dun et al. 2021). Other potatial targets, including the manipulation of the Auxin Response Factor (Bouzayen et al. 2013), and DELLA (Ariizumi et al. 2021), are also covered by patent applications (Table 3). One of the most promising targets, down-regulation of *AGL6* (Table 1) has been shown to result in seedless tomato fruit, and importantly, these fruits were described as being of nornal weight and shape (Klap et al. 2017; Takisawa et al. 2018), which is not always the case; however, use of *AGL6* is the subject of a patent application (Barg et al. 2021).

**Table 3:** Summary of the relevant patent (legal status denoted as active) and patent applications (denoted as pending) for targets identified in Tables 1 and 2 pertaining to the development of seedless and stoneless fruit

Patent number	Gene	Title	Applicants	Pub	Earliest priority	Legal status	Ref
US 10941411 B2	PIN4	Modified gene resulting in parthenocarpic fruit set	Rijk Zwaan Zaadteelt en Zaadhandel Bv	Mar 9, 2021	Jan 30, 2015	Active until 2036	Van Dun et al. (2021)
WO 2013 034722 A1	ARF	New parthenocarpic plants with modified expression of auxin response factors and the microRNAs inducing said modified expression	Institut National Polytechnique de Toulouse	Mar 14, 2013	Sep 7, 2011	Pending	Bouzayen et al. (2013)
US 2021 0037779 A1	AGL6	Parthenocarpic plants and methods of producing same	The State of Israel Ministry of Agriculture	Feb 11, 2021	Jan 21, 2016	Pending	Barg et al. (2021)
WO 2020 252167 A1	STK	Methods of producing plants with altered fruit development and plants derived therefrom	Pairwise Plants Services Inc	Dec 17, 2020	Jun 11, 2019	Pending	Crawford and Poorten (2020)
WO 2021 040011 A1	DELLA	Fruit-bearing plant exhibiting high temperature resistance, high yield, and parthenocarpy	Univ Tsukuba, Ibaraki, Japan	Mar 4, 2021	Aug 30, 2018	Pending	Ariizumi et al. (2021)

The earliest priority date is the date at which the patent application claims priority over any other applications filed after that date

When granted, patents may potentially restrict the use of specific technologies for the development of parthenocarpic cherry, restrict gene targets to induce parthenocpary using genome editing, or restrict access to the market in specific countries or regions. It should be stressed that patent applications often contain speculative or unsubstantiated claims and there is no certainty that the applications in this summary will be approved and thereby achieve the status of a granted patent.

## Conclusions

Seedless/stoneless cherry fruit would be highly advantageous to the cherry industry. First, it is well known that sweet cherry pollination is mediated by insects, which is important for the production of a viable crop (Eeraerts et al. 2020). Wild pollinators, including solitary bees, are essential to ensure sweet cherry yields (Eeraerts et al. 2019); however, growers rely on commercial domesticated honeybees (*Apis mellifera*) for pollination at a cost up to 1000 euro per hectare, a considerable investment for commercial cherry producers. The absence of the need for pollination also beings the potential for higher fruit yields and the absence of the seed/stone an increase in flesh content. With no need for pollination, the significant costs of providing commercial domesticated honeybees to carry out this function would no longer be required, thus not only reducing production costs but also the reliance on the presence of wild bee populations and solitary bees, which also play a considerable role in fruit set. Furthermore, reliance on honeybees also comes with the drawback that they remain inactive under unfavorable weather conditions, such as at temperatures below 12 °C or heavy rainfall, which can provide additional drawbacks to cherry production. Second, one of the possible causes of ‘June drop’ is poor or inadequate pollination, possibly due to a reduction in insect activity. Fruit set without pollination has the potential to mitigate June drop and increase and protect yields. Finally, there is also the suggestion that June drop occurs due to signals received by the plant from the aborted seed. In the absence of the seed, that signal is removed, and fruit previously dropped may develop to full maturity, thereby increasing cherry yield for growers and potentially reducing cost to the consumer. The absence of the seed has been reported to increase fruit quality (seed can have a hard texture and/or unpleasant flavour). Furthermore, seeds can release compounds that result in fruit spoilage, and therefore, the absence of the seed can increase shelf life. In such instances, a seedless fruit may still be advantageous to growers even if the stone remains; removal of the need for pollination increasing yields and the inhibition of June drop reducing yield loss. Seedless fruit has also been reported to reduce yield fluctuations in pepper (Heuvelink and Körner 2001).

In conclusion, there are a large number of factors involved in fruit set and the pathways are not linear, but there are regulation checkpoints at different levels, redundancy and crosstalk, which together make this process extremely complicated. There is still a lot that has to be learnt and constantly new proteins, pathways, and hormones are being found to have a function in this already complex scenario; these variables include the role of jasmonic acid and its derivatives (Schubert et al. 2019), SPOROCYELESS/NOZZLE (Rojas-Gracia et al. 2017), and also microRNAs (Wang et al. 2018). It should be noted that we have addressed the removal of the seed from cherry fruit in this review; however, the seed is contained within a lignified stone, which remains in the absence of the seed. The removal of the stone would be the next step in the breeding of a truly seedless/stoneless fruit fit for industrial exploitation.

### *Author contribution statement*

EV and AJS: wrote the first draft of the manuscript; ML, MC, and JMD: contributed to the revised version of the manuscript.

## Data Availability

Data sharing is not applicable to this article as no datasets were generated or analysed during the current study.
